# Role of the SLIT-ROBO signaling pathway in renal pathophysiology and various renal diseases

**DOI:** 10.3389/fphys.2023.1226341

**Published:** 2023-07-11

**Authors:** Li Feng, Hua-Pan Shu, Lu-Lu Sun, Yu-Chi Tu, Qian-Qian Liao, Li-Jun Yao

**Affiliations:** Department of Nephrology, Union Hospital, Tongji Medical College, Huazhong University of Science and Technology, Wuhan, China

**Keywords:** SLIT-ROBO, kidney, fibrosis, inflammation, podocytes

## Abstract

SLIT ligand and its receptor ROBO were initially recognized for their role in axon guidance in central nervous system development. In recent years, as research has advanced, the role of the SLIT-ROBO signaling pathway has gradually expanded from axonal repulsion to cell migration, tumor development, angiogenesis, and bone metabolism. As a secreted protein, SLIT regulates various pathophysiological processes in the kidney, such as proinflammatory responses and fibrosis progression. Many studies have shown that SLIT-ROBO is extensively involved in various aspects of kidney development and maintenance of structure and function. The SLIT-ROBO signaling pathway also plays an important role in different types of kidney disease. This article reviews the advances in the study of the SLIT-ROBO pathway in various renal pathophysiological and kidney disorders and proposes new directions for further research in this field.

## 1 Introduction

In terms of structure and function, the kidney is potentially one of the most complicated organs in the human body (Abed et al., 2015). Over 850 million people worldwide are affected by kidney disease, and frighteningly, chronic kidney disease is predicted to become the fifth largest cause of death in the world by 2040 (Foreman et al., 2018; Jager et al., 2019). The continued progression of kidney inflammation can lead to fibrosis, which can cause irreversible damage to the kidneys (Meng et al., 2014). In addition, the incidence of podocyte-related nephropathy is progressively increasing, so there is an urgent need to elucidate the underlying mechanisms that regulate the structure and function of glomerular podocytes.

The SLIT-ROBO pathway was first identified as an axon guide in nervous system development (Brose et al., 1999; Kidd et al., 1999; Li et al., 1999) and has since been shown to function in areas such as cardiac and vascular morphogenesis, bone metabolism, and tumoral development (Dallol et al., 2002; Guan et al., 2003; Dickinson et al., 2004; Zhou et al., 2011; Li et al., 2015; Rama et al., 2015).

In neurogenesis, SLIT acts as a chemorepulsive midline cue that binds to the receptor ROBO, expressed on axonal growth cones, to control midline crossing (Kidd et al., 1999). SLIT2 acts at an early stage of osteoclast development and binds directly to the ROBO1 receptors of preosteoclasts to inhibit osteoclast differentiation (Park et al., 2019). SLIT2 also reduces the migration and fusion of preosteoclast cells and thus inhibits osteoclast formation (Park et al., 2019). In osteoblasts, SLIT3 binds to ROBO1/2 on the cell membrane and activates *ß*-catenin, promoting osteoblast proliferation and migration and facilitating bone formation (Kim et al., 2018; Koh, 2018).

In the developing mouse heart, deletion of *Robo1* alone or both *Robo1* and *Robo2* results in the absence of a membranous ventricular septum at birth, a deletion also cause by the *Slit3* mutation (Mommersteeg et al., 2013). In addition, deletion of *Robo1* resulted in a partial absence of the pericardium (Mommersteeg et al., 2013). The role of SLIT2 in angiogenesis is highly context dependent. SLIT2 drives retinal angiogenesis and endothelial cell migration by binding to ROBO1 and ROBO2 receptors (Rama et al., 2015). In contrast, in systemic sclerosis (SSc), the SLIT2-ROBO4 interaction interferes with angiogenesis by inhibiting Src kinase phosphorylation (Romano et al., 2018). The differential roles of SLIT2 in angiogenesis may depend specifically on binding to ROBO1 or ROBO4 receptors and the differential regulation of downstream signaling to endothelial cells.

The SLIT-ROBO pathway plays a dual role in cancer, promoting tumor progression in pancreatic (Han et al., 2015), nasopharyngeal (Alajez et al., 2011) and prostate cancers (Latil et al., 2003), and acting as a cancer suppressor in gastric, lung, breast and ovarian cancers (Dickinson et al., 2011; Lahoz and Hall, 2013; Wen et al., 2014; Zhang et al., 2015).

This paper provides a review of the research advances in the SLIT-ROBO signaling pathway in various physiological and pathological processes in the kidney, aiming to shed new light on the prevention and management of kidney disorders.

## 2 Structure and characteristics of SLIT proteins

SLIT is a highly conserved secreted protein containing approximately 1,500 amino acids (Dickson and Gilestro, 2006). It was identified for the first time in a genetic screen in *Drosophila* and was found to be involved in midline axonal repulsion during the development of the central nervous system (Simpson et al., 2000).The *Slit* gene and its expression were subsequently detected in *Caenorhabditis elegans* (Hao et al., 2001), chickens (Hao et al., 2001; Holmes and Niswander, 2001), *Xenopus* (Li et al., 1999; Hao et al., 2001), rats (Itoh et al., 1998; Yi et al., 2006), mice (Brose et al., 1999; Hao et al., 2001), and humans (Itoh et al., 1998; Hao et al., 2001). The SLIT family has three subtypes in mammals, namely, SLIT1, SLIT2, and SLIT3 (Dickinson et al., 2004; Fujiwara et al., 2006).The *SLIT1* gene is located on human chromosome 10q24.1, the *SLIT2* gene is located on 4p15.31, and the *SLIT3* gene is located on 5q34-q35.1 (Katoh and Katoh, 2005). Initial studies reported that SLIT1 was mainly expressed in the nervous system, and as research progressed, SLIT2 and SLIT3 were detected in the nonneural system (Brose et al., 1999). According to available studies, the SLIT protein is expressed in brain, lung, pancreatic, and breast cancer tissues, as well as in macrophages, neutrophils, glomerular endothelial cells, and mesangial cells (Gu et al., 2015; Fan et al., 2016; Ding et al., 2020).

**FIGURE 1:**
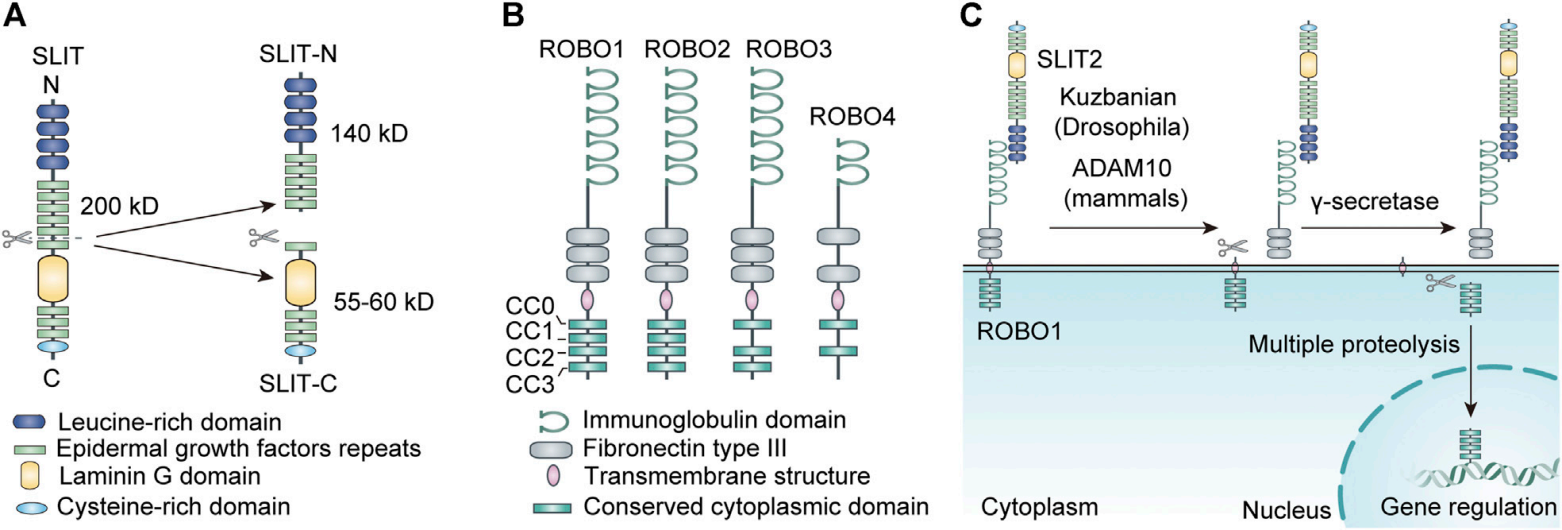
**(A)** The structure and proteolysis of SLIT. **(B)** The structure of ROBO. **(C)** The proteolysis of ROBO.

The SLIT family has a high degree of homology among species (Dickinson et al., 2004). Structurally, it consists of a putative signal peptide, four leucine-rich repeats (LRRs), 7 (*Drosophila* SLIT) to 9 (vertebrate SLIT) epidermal growth factor (EGF) repeats, a laminin G domain, and a cysteine-rich domain (Rothberg et al., 1990; Rothberg and Artavanis-Tsakonas, 1992) (Figure 1). Most SLIT proteins can be fragmented by an unknown protease between the fifth and sixth EGF repeats, with a conserved proteolytic site (Brose et al., 1999; Wang et al., 1999; Patel et al., 2001; Kellermeyer et al., 2020).

According to existing studies, different SLIT fragments and SLIT subtypes have distinct functions *in vivo*. Research has shown that SLIT2 is an ∼200kD glycoprotein generally cleaved into a 55-60kD C-terminal segment (SLIT2-C), a 140kD N-terminal segment (SLIT2-N), and some unknown segments (Nguyen Ba-Charvet et al., 2001) (Figure 1). The whole-length SLIT2 and SLIT2-N-terminus both combine with ROBO receptors to repel axons, neurons, and leukocytes such as neutrophils; promoted neural system development; and exhibit anti-inflammatory effects. The junction of SLIT2-C with the cell membrane is less tight than that with SLIT2-N. SLIT2-C does not bind to ROBO receptors but can bind to the semaphorin receptor PlexinA1 to act as a commissural axon guide (Delloye-Bourgeois et al., 2015). Similar to SLIT2, SLIT3 can also be degraded into C-terminal and N-terminal fragments by proteases. A study found that the N-terminal fragment of recombinant SLIT3 can interact with ROBO4 during angiogenesis to promote angiogenesis. Thus, mice lacking SLIT3 or with *Slit3* mutations may present disruption in the development of diaphragmatic vessels, leading to diaphragmatic herniation (Zhang et al., 2009; Zhang et al., 2014). In addition, recent studies have found evidence that the C-terminal portion of recombinant hSLIT3 can conjugate with both heparin and heparan sulfate, effectively neutralizing the anticoagulant activity of heparin and exerting the detoxifying effect of excess heparin and heparan sulfate (Condac et al., 2012).

## 3 Structure and characteristics of ROBO proteins

ROBO is the predominant receptor for the SLIT ligand family. It is a single-pass transmembrane receptor protein with a conserved intracellular domain and consists of 1,000–1,600 amino acids (Jiang et al., 2022). Similar to the SLIT family, ROBO was first discovered in a *Drosophila* genetic screen (Seeger et al., 1993). Although ROBO receptors are strongly conserved, the quantity of *Robo* genes varies slightly among species. There is only one ROBO receptor in Xylia; three ROBO receptors (ROBO1, ROBO2, and ROBO3) in *Xenopus*, chicken, and *Drosophila*; and four ROBO receptors (ROBO1/DUTT1, ROBO2, ROBO3/RIG-1, and ROBO4/Magic Roundabout) are found in mammals such as humans (Huminiecki et al., 2002; Hohenester, 2008). In human chromosomes, the *ROBO1* and *ROBO2* genes are at 3p12.3, and the *ROBO3* and *ROBO4* genes are at 11q24.2 (Tong et al., 2019).

ROBO transmembrane receptors are immunoglobulin superfamily members with cell adhesion molecules (Hohenester, 2008). ROBO1-3 are displayed in cells of various tissues, especially in the neural system. ROBO1-3 share a similar protein structure, made up of five immunoglobulin (Ig) structural domains, three fibronectin type III (FN III) repeat sequences, a transmembrane structure, and an intracellular structural domain (Evans and Bashaw, 2010; Wang and Ding, 2018). The cell cytoplasmic structural domains of ROBO1 and ROBO2 encompass four well-conserved proline-rich structural domains, named CC0, CC1, CC2, and CC3; the cytoplasmic domain of ROBO3 has only three domains, CC0, CC2, and CC3 (Wang and Ding, 2018). ROBO4 is mainly expressed in the vascular endothelium and is involved in angiogenesis (Park et al., 2003; Bedell et al., 2005). The structure of ROBO4 is dissimilar to that of the other ROBO receptors. Its external structure contains only two Ig structural domains and two FN3 structural domains, while the intracellular part consists of only the CC0 and CC2 structural domains (Huminiecki et al., 2002) (Figure 1). The intracellular structural domains of ROBO receptors have no fixed catalytic activity. However, they can exert their function by recruiting or activating downstream signaling molecules through the proline-rich structural domains described above (Dickson and Gilestro, 2006).

Interestingly, the ROBO family can also be cleaved into different fragments by a variety of metalloproteases at the posttranscriptional level, which dramatically increases its functional diversity. It has been reported that SLIT ligands located in the extracellular matrix bind to the extracellular structural domain of the ROBO receptor, which leads to exposure of the metalloprotease scission site in the near-membrane area of the ROBO protein, resulting in cleavage of the ROBO receptor (Coleman et al., 2010). The extracellular component of the ROBO receptor is lost after cleavage. In addition, it was found in human tumor cells that the carboxyl terminus of the ROBO protein, which is the intracellular structure produced by metalloprotease cleavage, can be further fragmented by *γ*-secretase, and the resulting smaller C-terminal segment ROBO protein can translocate to the nucleus, leading to nuclear accumulation of the ROBO C-terminal fragment (Seki et al., 2010). The carboxyl terminus of some transmembrane receptors is also trafficked to the nucleus after multiple proteolyses to mediate target gene transcription (Fortini, 2002). The intracellular structure of the ROBO receptor can combine with a variety of molecules to deliver signals (Figure 1). It is unclear whether this nuclear translocation phenomenon affects signaling downstream of ROBO.

## 4 SLIT-ROBO signaling pathway

The second LRR domain of the SLIT protein binds to the active site in the first Ig domains of ROBO1 and ROBO2 to transmit cellular signals. In mammals, alteration of several amino acids in the first Ig domain of ROBO3 prevents it from binding to SLIT (Zelina et al., 2014). The binding residues in SLIT2 required for ROBO1 and 2 binding are lost in ROBO4 (Koch et al., 2011).Biochemistry and genetics studies have found that heparan sulfate (HS) proteoglycan is necessary for SLIT-ROBO signaling and that it is an indispensable coreceptor in SLIT-ROBO signals. The presence of HS can not only stabilize the SLIT-ROBO complex by binding to the SLIT ligands of the extracellular matrix but also assist the signaling of ROBO receptors and mediate the biological effects of cells (Steigemann et al., 2004).

SLIT ligand and ROBO receptor binding are the most common but not the only mode of binding. There is increasing evidence that SLIT and ROBO proteins can bind to other receptors and ligands, respectively, and mediate different biological roles. The N-terminus of SLIT can combine directly with the N-terminal Ig domain of Dscam1, promoting the association of Dscam1 with receptor tyrosine phosphatase69D (RPTP69D), which in turn causes the direct dephosphorylation of Dscam1 (Dascenco et al., 2015). This feature of SLIT is unrelated to ROBO receptors and electively promote the extension of specific axon collaterals (Dascenco et al., 2015). SLIT-C can specifically attach to the semaphorin receptor PlexinA1 to exert a coaxonal guiding role (Delloye-Bourgeois et al., 2015); In addition, SLIT-C also regulates the spatial distribution of cells by binding to the basement membrane scaffold protein dystroglycan (Wright et al., 2012). Recent studies have found that the C-terminus of SLIT2 activates the PKA signaling pathway and adjusts glucose homeostasis (Svensson et al., 2016).

The SLIT protein has been thought to be the only ligand for the ROBO receptor. However, several amino acid changes in the first Ig of ROBO3 cause it to lose the ability to bind to the SLIT family but allow it to bind with NELL ligands (Zelina et al., 2014). The neural epidermal growth factor (NELL) protein was found to be a ligand for ROBO2/3, but not ROBO1/4. Interaction analysis with NELL1 and ROBO receptor families revealed that NELL1/2 is capable of binding to the first FN III domain of ROBO2, but this binding relies on a conformational transition in the ROBO2 protein that discloses its binding site to NELL (Barak et al., 2019; Yamamoto et al., 2019).In addition, NELL2 binds to ROBO3 to inhibit SLIT2-ROBO1/2-mediated repulsion and guide axons across the midline (Jaworski et al., 2015). The more mysterious ROBO4 receptor has not been found to have a direct interaction with the SLIT family, ROBO4 has been found to interact with another guidance receptor Unc5B and to regulate angiogenesis independently of its intracellular domain (Koch et al., 2011; Zhang et al., 2016). Hence, more research on ROBO4 is needed. The SLIT-ROBO signaling pathway and interactions with other related ligand receptors significantly add to the variety and sophistication of the pathway.

## 5 SLIT-ROBO function in the kidney

### 5.1 SLIT-ROBO and kidney development

#### 5.1.1 SLIT-ROBO and congenital anomalies of the kidney and urinary tract

A series of malformations afflicting kidney morphogenesis and other components of the urinary tract are collectively referred to as congenital anomalies of the kidney and urinary tract (CAKUT) (Westland et al., 2020). The pathogenesis of CAKUT has not been fully elucidated, but abundant investigations suggest that single gene abnormalities are relevant to the development of the disease (Kanda et al., 2020; Saygili et al., 2020; Wang et al., 2020; Wu et al., 2020; Li Y. et al., 2021).In a large cohort study, *ROBO2* gene mutations were strongly linked to congenital renal and urethral abnormalities, particularly vesicoureteral reflux (VUR) (Hwang et al., 2014). In another study, researchers investigated a male with a novel translocation, 46, X,t (Y;3) (p11;p12)dn, who exhibited various congenital anomalies, including severe bilateral ureteral ligation defects. This translocation impairs the *ROBO2* gene, which encodes the transmembrane receptor for the SLIT ligand, ROBO2, reducing ROBO2 protein expression (Lu et al., 2007). At the same time, they found that adult heterozygous mice with decreased *Robo2* gene dose also presented a distinct CAKUT-VUR phenotype (Lu et al., 2007).

Bertoli-Avella et al. investigated the possible causal relationship of variants in the *ROBO2* gene in 95 individuals with native VUR or unrelated VUR/CAKUT. They identified 24 variants of the *ROBO2* gene, four amino of, which resulted in acid replacements: Asp766Gly, Arg797Gl, Asn515Ile, and Gly328Ser. [15] Another study showed that SLIT2 and its receptor ROBO2 function as key players in the formation of ureteral buds. Mutations in *SLIT2* and *ROBO2* cause ureteral dysplasia, the development of multiple ureteral buds, and improper insertion of the ureter into the renal duct, leading to the reflux of urine. [75] The interaction of the ureteral bud and metanephric mesenchyme is critical for renal development. [76, 77] SLIT-ROBO signaling restricts the extent of nephrogenic regions by restricting epithelial/mesenchymal mutual effects in the neonatal metanephric kidney (Wainwright et al., 2015).

A recent study analyzed the genetic burden of 26 unresolved CAKUT families after whole-exome sequencing. They found two heterozygous mutations in *SRGAP1* in two unrelated families. SRGAP1 is essential for metanephric development and is a GTPase-activating protein of the SLIT2-ROBO2 signaling pathway. Further studies suggest that a gain-of-Function mutation in *SRGAP1* and a loss-of-Function mutation in *SLIT2* may cause innate anomalies in the kidney and urinary tract (Hwang et al., 2015). Thus, the SLIT2-ROBO2 signaling pathway and its downstream regulators have been demonstrated to be engaged in the development of congenital abnormalities of the nephron and urethra.

#### 5.1.2 SLIT-ROBO and renal angiogenesis

In the vascular system, SLIT2 is known to play a role in promoting angiogenesis through ROBO1 and ROBO2 on endothelial cells. Mechanistically, it promotes endothelial cell migration by activating the Rac1 signaling pathway transduction. Additionally, ROBO1/2 is required for a vascular endothelial growth factor (VEGF)-induced activation of Rac1 and lamellipodia formation (Rama et al., 2015).

Recent studies have revealed that the SLIT2-ROBO signaling pathway plays a crucial role in the formation of the glomerular capillary plexus (Li J. et al., 2021). One study demonstrated that alkaline-phosphatase–conjugated full-length SLIT2 protein (SLIT2-FL-AP) binds to glomerular capillaries but not to other renal vessels, suggesting that SLIT2 has a specific role in the glomerulus (Li J. et al., 2021). In contrast to Cre-negative littermates, global deletion of *Slit2* (*Slit2* ^*f/f*^ *; Rosa-Cre* ^*ERT2*^) in neonatal mice with small kidney volumes reduced the numbers of vascularized glomeruli in the superficial cortex, without significant changes in vascularized glomeruli in the juxtamedullary region (Li J. et al., 2021). In terms of mechanism, SLIT2 may promote glomerular angiogenesis by stimulating endothelial cell proliferation and migration, as glomerular endothelial cell proliferation is reduced and apoptosis is increased in SLIT2^iKO^ mice (Li J. et al., 2021). Moreover, in SLIT2^iKO^ mice, the VEGF signaling pathway, which is essential for glomerular angiogenesis, was inhibited and protein blots showed a significant reduction in VEGFR2 expression in kidney tissue (Li J. et al., 2021). This suggests that in the absence of SLIT2, defective VEGFR2 signaling leads to defects in glomerular vascularity. In addition, some other evidence suggests that SLIT2-ROBO1/2 can link VEGFR2 to Endophilin-A2 (ENDO2), a BAR-structured protein that coordinates CLATHRIN-independent internalization and promote VEGFR2 internalization and downstream signaling cascades, thereby stimulating endothelial cell migration and angiogenesis (Genet et al., 2019). In conclusion, deletion of SLIT2 not only reduces VEGFR2 expression but also inhibits VEGF signaling by suppressing VEGFR2 internalization.

ROBO1,2^iKO^ mice exhibited a similar phenotype of reduced endothelial cells in the glomerulus, whereas ROBO4^−/−^ mice did not, suggesting that SLIT2 promotes glomerular vascular development primarily through ROBO1/2. Mice with endothelial-specific *Robo2* deletion (*Robo1* ^*−/−*^ *; Robo2* ^*f/f*^ *; Cdh5-Cre* ^*ERT2*^) also exhibited reduced glomerular endothelial cells, suggesting that SLIT2 acts, at least in part, through the ROBO1/2 receptor in glomerular endothelial cells (Li J. et al., 2021). This study suggests for the first time that SLIT2-ROBO signaling plays a crucial role in glomerular angiogenesis and suggests a positive therapeutic role for SLIT2 as a novel specific regulator of glomerular angiogenesis in a range of diseases that cause severe glomerular vascular loss.

### 5.2 SLIT-ROBO and renal pathophysiology

#### 5.2.1 SLIT-ROBO and kidney inflammation

Studies have shown that SLIT2 effectively inhibits TNF-a-induced neutrophil adhesion to human umbilical vein endothelial cells (HUVECs) (Chaturvedi et al., 2013). SLIT2 mediates anti-chemotaxis by blocking chemoattractant-induced cell polarization ad actin barbed end formation (Tole et al., 2009). Importantly, this effect of SLIT2 depends on its receptor ROBO1 (Prasad et al., 2007; Tole et al., 2009; Chaturvedi et al., 2013), which was demonstrated to be expressed in inflammatory cells such as neutrophils, dendritic cells, and macrophages (Guan et al., 2003; Prasad et al., 2007; Tole et al., 2009; Chaturvedi et al., 2013; Geraldo et al., 2021; Yusuf et al., 2021) (Table 1).

**TABLE 1:** Downstream regulation and role of the SLIT-ROBO signaling pathway in various cells.

Cell type	SLIT/ROBO pathway	Downstream signaling molecules	Function	References
Neutrophil	SLIT2-ROBO1	Inhibit Rho GTPases, Rac2 and Cdc42	Inhibit neutrophil chemotaxis	Tole et al. (2009)
				Chaturvedi et al. (2013)
T cells	SLIT2-ROBO1	Inhibit Src, Lck, Akt, Rac	Inhibit the CXCL12/CXCR4-induced chemotaxis of T cells	Prasad et al. (2007)
Langerhans cells	SLIT2-ROBO1	None	Inhibit the migration of Langerhans cells	Guan et al. (2003)
Macrophages	SLIT2-ROBO1	Activate RhoA	Inhibit macrophage spreading and induce cell rounding	Bhosle et al. (2020)
Monocytes	SLIT2-ROBO1	Inhibit Rho GTPases, Rac1 and Cdc42 and the activity of PI3K and Erk1/2	Inhibits monocyte adhesion to endothelial cells	Mukovozov et al. (2015)
HUVECs	SLIT2-ROBO1/2	Activate Rac1	Promote retinal angiogenesis	Rama et al. (2015)
MVECs	SLIT2-ROBO4	Inhibit Src	Inhibit angiogenesis	Romano et al. (2018)
HUVECs	SLIT2-ROBO4	Inhibit Pyk2	Inhibit LPS-induced endothelial inflammation	Zhao et al. (2014)
NRK49F	SLIT2-ROBO1	Inhibit RhoA	Inhibits TGF-β–induced fibroblast activation and renal fibrosis	Yuen et al. (2016)
Podocytes	SLIT2-ROBO2	ROBO2 interacts with Nck to inhibit nephrin	Inhibits nephrin-induced actin polymerization	Fan et al. (2012)
Podocytes	SLIT2-ROBO2	ROBO2/SRGAP1/NMIIA form a complex; inhibited NMII activity	Reduced formation of focal adhesion	Fan et al. (2016)

Further studies revealed that SLIT2 impacts neutrophil chemotaxis in addition to other crucial stages of neutrophil recruitment, such as trapping, adherence, and transendothelial translation. Another study found that SLIT2 was upregulated in the skin by allergen sensitization and downregulated Langerhans cell migration through direct interaction with ROBO1, which in turn led to inhibition of contact hypersensitivity (Guan et al., 2003). Furthermore, investigations by Prasad et al. found that SLIT2 inhibited CXCL12-mediated T-cell and monocyte transendothelial migration and chemotaxis (Prasad et al., 2007). Overall, some of the above findings suggest the possibility that SLIT2 may play a fundamental role in suppressing inflammatory responses, and anti-inflammatory therapy targeting the SLIT2-ROBO1 pathway may be promising.

However, some studies have also found that ROBO1 may be proinflammatory, as ROBO1 downregulation suppresses LPS-induced cytokine expression in HUVECs (Zhao et al., 2014). This study suggested that SLIT2 inhibits LPS-induced chemokine and inflammatory cytokine production, upregulaing the cell attachment protein ICAM-1, and monocyte adherence through the endothelium-specific receptor ROBO4 (Zhao et al., 2014).

#### 5.2.2 SLIT-ROBO and kidney fibrosis

Inflammation is a predisposing factor for fibrosis (Kuusisto et al., 2012; Wong et al., 2014; Hartupee and Mann, 2016; Anzai, 2018; Mack, 2018). Tissue damage and inflammatory reactions result from various causes (Fleming et al., 2010; Oishi and Manabe, 2018). The injured tissue secretes several chemokines and cytokines, the most important of which are TNF-α and IL-8. The secreted cytokines recruit various immune cells to accumulate at the site of the injury (Son et al., 2014; Zheng et al., 2019). These immune cells secrete profibrotic mediators, such as TGF-β, which induce fibrosis to promote tissue repair and healing (Lan, 2011; Conti et al., 2018). The inflammatory response and fibrosis progression complement each other and are jointly responsible for tissue damage. Recurrent long-term inflammation, damage and repair of tissues, and hyperaccumulation of the extracellular matrix (ECM) can lead to renal insufficiency and eventually unreversible glomerulosclerosis. Because fibrosis causes irreversible damage to kidney tissue, early intervention before progression to fibrosis is particularly critical.

The secretory ligand SLIT2 and its receptor ROBO1 have been shown to manage the migration of cells, including smooth muscle cells, macrophages, neutrophils, T lymphocytes, dendritic cells, and perivascular cells (Wu et al., 2001; Guan et al., 2003; Liu et al., 2006; Prasad et al., 2007; Tole et al., 2009; Guijarro-Muñoz et al., 2012; Chaturvedi et al., 2013; Pilling et al., 2014; Chen et al., 2021; Geraldo et al., 2021). Cell migration involves rearrangement of the cytoskeleton (Adamson et al., 1999). Previous studies have reported that SLIT2 inhibits the activation of Rac2 and CDC42, which is induced by chemotactic agents in neutrophils (Tole et al., 2009). Upon the action of chemokines, activation of the small Rho GTPase family members Rac1, Rac2, CDC42, and RhoA plays a pivotal role in the rearrangement of cytoskeletal actin (Wong et al., 2001; Liu et al., 2006; Filippi et al., 2007). These individuals of the small Rho GTPase family are important mediators of cytoskeletal rearrangements, an important physiological change in cell migration (Nobes and Hall, 1995; Hall, 1998; Raftopoulou and Hall, 2004; Filippi et al., 2007). Notably, the progression of fibrosis also involves cytoskeletal rearrangements (Buckley et al., 2012). The well-known profibrotic cytokine TGF-β activates multiple cell types to differentiate into myofibroblasts that synthesize collagen and the extracellular matrix (Willis and Borok, 2007; Zhang et al., 2019). The combination of TGF-β and its receptors induces the phosphorylation of Smad2 and Smad3 intracellular structures on serine residues. Phosphorylated Smad2 and Smad3 bind to Smad4 and then enter the nucleus, where Smad proteins induce the expression of profibrotic genes (Attisano and Wrana, 2002). Smad protein phosphorylation is cytoskeleton dependent, and pharmacological disruption of the actin cytoskeleton significantly reduces basal and TGF-β1-mediated increases in collagen I and IV (Attisano and Wrana, 2002). Likewise, the Rho-family GTPase suppressor *Clostridium difficile* toxin B or the Rho-related kinase suppressor Y-27632 stopped the action of TGF-β1 (Attisano and Wrana, 2002).

**FIGURE 2:**
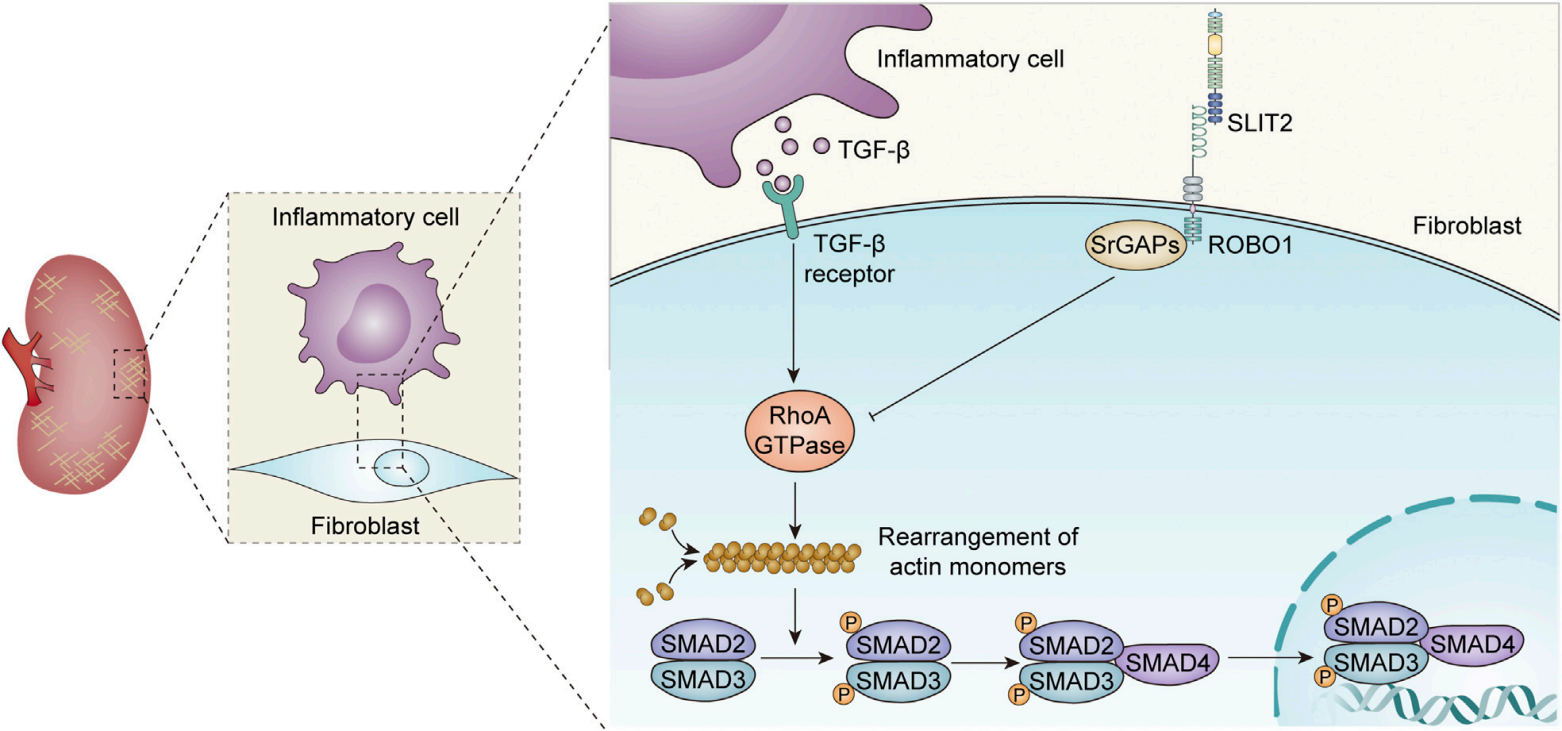
Role of the SLIT-ROBO signaling pathway in renal fibrosis.

The SLIT2-ROBO1 signaling pathway is likely to affect the rearrangement of cytoskeletal actin by inhibiting the activation of small Rho GTPase family members, thereby improving fibrosis (Figure 2). The study by Yuen et al. found that SLIT2 could inhibit the formation of actin stress fibers and TGF-β-mediated Smad signaling, delay the progression of renal fibrosis, and improve renal function, which is mainly reflected in the decrease in serum creatinine levels (Yuen et al., 2016). A recent study showed that SLIT2 ameliorated kidney fibrosis and inflammation after hypoxia and LPS-induced epithelial cell injury by impacting the TLR4/NF-κB pathway in the renal tubules, a process that was independent of HIF-1α (Zhou et al., 2017).

The SLIT2-ROBO signaling pathway has different effects on fibrosis in different cell types and different disease models (Pilling et al., 2014; Huang et al., 2020; Ahirwar et al., 2021; Liu et al., 2021).Fibroblast-secreted SLIT2 inhibits monocyte-to-fibroblast differentiation, and rSLIT2 injection reduces bleomycin-induced pulmonary fibrosis (Pilling et al., 2014). Similar studies have shown that endogenous SLIT2 restrains TGF-β1-mediated compliance signaling in lung fibroblasts (Huang et al., 2020). Importantly, recent studies have found that SLIT2 reduces fibrosis in mice with breast cancer by enhancing the production of metalloproteinase 13 expression in tumor-resistant M1-like macrophages (M1-TAMs) (Ahirwar et al., 2021). However, significantly increased collagen I and α-smooth muscle actin (α-SMA) production were found in SLIT2-Tg mice, suggesting that SLIT2-ROBO1 pathway activation contributes to liver fibrosis (Chang et al., 2015; Li C. et al., 2019). Another study discovered that rSLIT2 treatment dramatically raised the expression of profibrotic mediators and extracellular matrix components in mouse hepatic stellate cells, suggesting that SLIT2-ROBO2 transduction facilitates liver fibrosis (Zeng et al., 2018). Consistently, the SLIT2-ROBO1 signaling also promotescardiac fibrosis, which is partially dependent on PI3K/AKT pathway activation (Liu et al., 2021). In addition, SLIT3, another isoform of the SLIT family, can be secreted by fibroblasts. SLIT3 deficiency inhibits fibroblast activity, reduces collagen type I expression, and ameliorates stress-induced cardiac fibrosis (Gong et al., 2020). In conclusion, although SLIT2-ROBO signaling plays different roles in fibrosis in other organs and kidney tissue, all current findings point to the SLIT2-ROBO1 pathway inhibiting renal fibrosis and improving renal function (Zhou et al., 2017).

### 5.3 SLIT-ROBO and homeostasis of the renal filtration membrane

**FIGURE 3:**
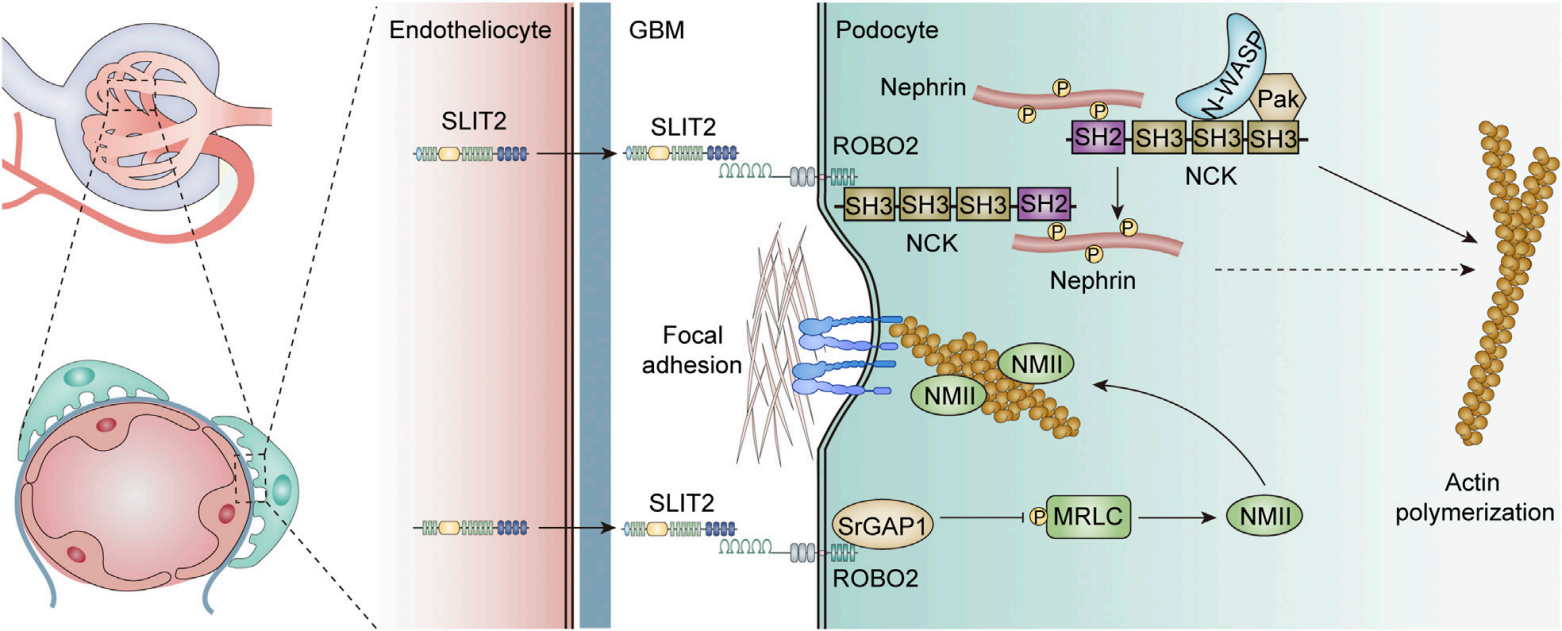
Role of the SLIT-ROBO signaling pathway in the glomerular filtration barrier.

In the glomerulus, ROBO2 and SRGAP1 are highly expressed in developing podocytes and in the basal surface of mature podocytes, while glomerular endothelial and mesangial cells secrete SLIT2 and SLIT3 (Fan et al., 2012; Fan et al., 2016). Genetic mutations in actin-related proteins induce foot process disappearance and proteinuria, suggesting that dynamic activity of the actin cytoskeleton is engaged in the generation and sustainment of complex foot process constructs (Krolewski and Bonventre, 2012). Notably, the SLIT-ROBO pathway regulates the rearrangement of the actin cytoskeleton by inhibiting the activity of the small Rho GTPase protein (Tole et al., 2009) (Figure 3). This implies that the SLIT-ROBO2 signaling pathway may play key roles in the homeostasis of network signaling in glomerular endothelial cells, mesangial cells, and podocytes, as well as the formation and maintenance of podocyte shape and function.

#### 5.3.1 SLIT-ROBO and podocyte foot process formation

In addition, the SLIT2-ROBO2 pathway has been demonstrated to be engaged in the formation of the podocyte foot process (Fan et al., 2012) (Figure 3). Nephrin (Nph) is an important slit-diaphragm protein, an adhesion molecule belonging to the immunoglobulin superfamily, used to link the foot processes of adjacent podocytes (Ruotsalainen et al., 1999). Nephrin knockout resulted in specific features of abnormal foot process widening, septum vanishing, and basal membrane thickening (Doné et al., 2008). The phosphorylated intracellular domain of nephrin recruits the SH2 domain at the C-terminus of the adaptor protein Nck and induces the SH3 domain at the N-terminus of Nck to recruit multiple cytoskeletal modulators, including Pak and N-WASP to induce actin polymerization (Jones et al., 2006; Verma et al., 2006). The ROBO2 receptor directly binds to the SH3 domain at the N-terminus of the adaptor protein Nck and forms a compound with nephrin via Nck to depress the actin-inducing effect of nephrin. Podocyte-specific ROBO2-deficient mice exhibited an irregular and disorganized staggered pattern of foot processes at approximately 4 weeks, further demonstrating the critical role of the ROBO2 protein in podocyte foot process formation. Interestingly, the knockdown of ROBO2, a negative regulator of nephrin, also ameliorated the abnormal podocyte phenotype caused by nephrin deletion (Fan et al., 2012). Although the exact mechanism by which ROBO2 mediates the actin-induced inhibition of Nephrin is unclear, it may involve competitive binding of the SH3 domain, and the mutual checks and balances of ROBO2 and nephrin together regulate podocyte foot process plasticity, supporting the stability of the glomerular filtration membrane.

#### 5.3.2 SLIT-ROBO in podocyte adhesion

The SLIT2-ROBO2 signaling pathway can not only interfere with the formation of normal foot processes in podocytes by inhibiting the function of nephrin but also reduce podocyte adhesion to the extramural matrix, resulting in the shedding and loss of podocytes from the basement membrane. Integrin α3/β1, a common and important adhesion molecule, binds to laminin-α5β2γ1 (laminin-521) on the glomerular basement membrane, forming a focal adhesive compound and maintaining the structural stability of the filtration membrane by connecting the cytoskeleton via several actin proteins (Sachs and Sonnenberg, 2013) (Figure 3). RhoA, a downstream regulator of the SLIT-ROBO pathway, has been proven to mediate stress fiber generation and actin polymerization by inducing rho-associated protein kinase (ROCK)-dependent phosphorylation of myosin II (Vicente-Manzanares et al., 2009). Nonmuscle myosin II (NMII) consists of two heavy chains, two regulatory light chains (MRLCs), and two essential light chains (MELCs), and is responsible for the generation of stress fibers in podocytes (Vicente-Manzanares et al., 2009). The SLIT2-ROBO2 signaling pathway interfered with NMII activation, suppressed stress fiber generation in podocytes, reduced the formation of focal adhesive, and reduced the adhesion of podocytes to the extracellular matrix. It has been reported that in the presence of SLIT2, MRLCs can directly interact with SLIT-ROBO Rho GTPase activating protein 1 (SRGAP1) and form a ROBO2-SRGAP1-NMII complex via MRLCs (Fan et al., 2016). Therefore, the SLIT2-ROBO2 pathway serves a nonnegligible function in the glomerular filtration screen, which involves not only the stabilization of podocyte structure but also the attachment of podocytes to the glomerular basement membrane.

### 5.4 SLIT-ROBO and renal disease

#### 5.4.1 Acute kidney injury

As previously described, SLIT2 is effective in inhibiting the migration and recruitment of leukocytes *in vitro* and exerts anti-inflammatory effects. This raises the question of whether SLIT2 also plays an important role in acute kidney injury, which also involves massive infiltration of inflammatory cells. Previous researchers have used anti-glomerular basement membrane (GBM) antibodies to induce a crescentic glomerulonephritis (GN) model in male Wistar-Kyoto (WKY) rats to investigate the role of SLIT2 in inflammation *in vivo* (Kanellis et al., 2004). This study demonstrated that the mRNA levels of glomerular *Slit2* were significantly reduced in induced crescentic glomerulonephritis (Kanellis et al., 2004). They injected rabbit polyclonal anti-SLIT2 antiserum into rats with glomerulonephritis and found that rats treated with anti-SLIT2 antiserum developed proteinuria and crescentic bodies earlier than control rats (Kanellis et al., 2004). In addition, injection of exogenous recombinant human SLIT2 (rhSLIT2) into crescentic GN rats significantly improved the histological and functional parameters of the rat kidney (Kanellis et al., 2004). Mechanistically, rhSLIT2 reduced the migration of leukocytes induced by the chemotactic inducer MCP1. This effect may be due to the inhibition of GTPase activity.

**FIGURE 4:**
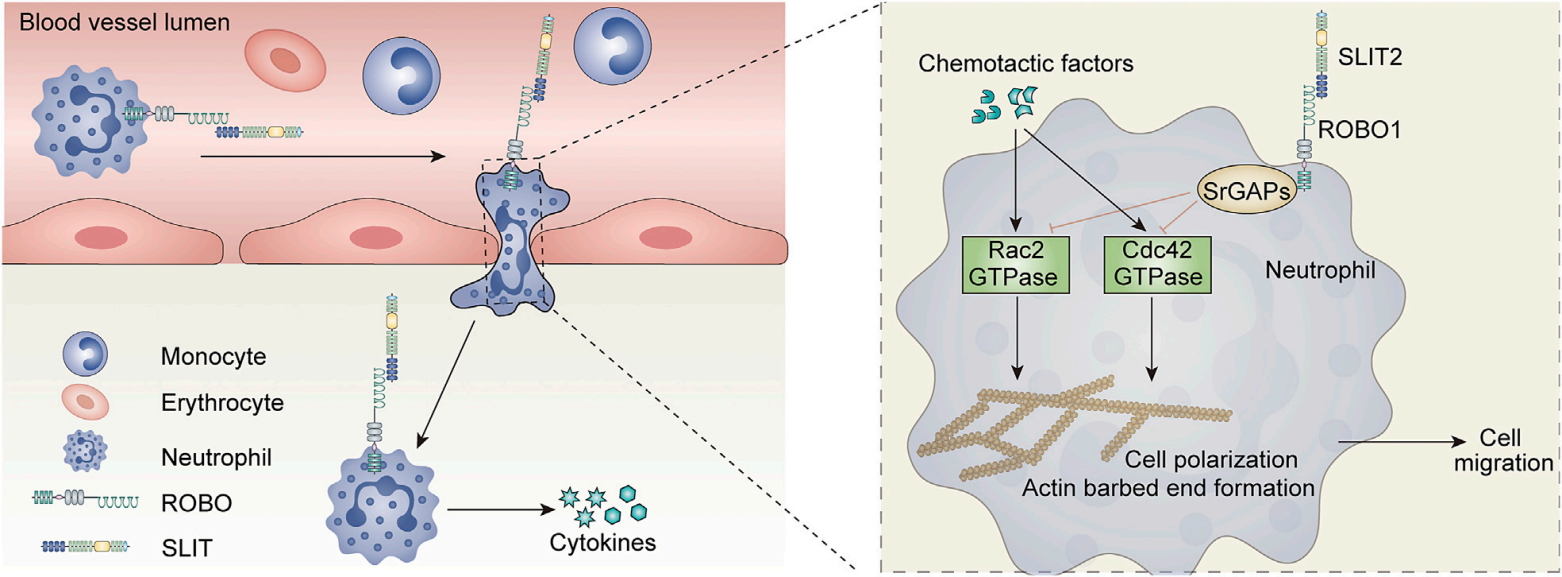
Role of the SLIT-ROBO signaling pathway in renal inflammatory response.

In another study, a mouse ischemia‒reperfusion injury (IRI) model was constructed using bilaterally clamped renal pedicles, and the protein levels of endogenous SLIT2 were significantly reduced in the renal IRI model (Chaturvedi et al., 2013). Creatinine levels in mice with renal IRI were significantly reduced by intraperitoneal injection of whole-length human SLIT2 (hSLIT2) and a bioactive N-terminal fragment of mouse SLIT2 (N-mSLIT2) (Chaturvedi et al., 2013). In contrast, the nephroprotective effect of N-mSLIT2 was markedly diminished after preincubation with the ROBO-1 receptor, suggesting that the nephroprotective effect of SLIT2 is dependent on the ROBO1 receptor (Chaturvedi et al., 2013). Mechanistically, SLIT2 reduces neutrophil adhesion and transendothelial cell migration to vascular endothelial cells exposed to hypoxia-reoxygenation (H/R) (Chaturvedi et al., 2013) (Figure 4).This study also showed that this inhibition of neutrophil adhesion was dependent on ROBO1 receptors on neutrophils rather than ROBO receptors on vascular endothelial cells (Chaturvedi et al., 2013). Neutrophils, immune cells in the body, also play an indelible role in innate immunity. Interestingly, although the SLIT2 protein inhibited chemokine-induced neutrophil migration, it did not impair protective neutrophil immune functions such as phagocytosis and superoxide production (Chaturvedi et al., 2013). Previous studies have shown that SLIT2 inhibits the migration of leukocytes through the regulation of GTPase activity (Kanellis et al., 2004). The varying effects of SLIT2 on different neutrophils may be due to the complex and unknown role of GTPases in these effects.

#### 5.4.2 Diabetic nephropathy

Podocytes play a vital role in maintaining glomerular filtration barrier function through their interdigitated foot processes. Disruption of the structural function of podocytes leads to the development of proteinuria. The previous section described how SLIT-ROBO signaling reduces F-actin polymerization via SRGAP1, thereby destabilizing podocytes (Fan et al., 2016). However, a different perspective was recently presented in studies on diabetic nephropathy, where the mRNA levels of SLIT-ROBO GTP activating protein 2a (SRGAP2a) were significantly reduced in patients with diabetic nephropathy (Pan et al., 2018). Furthermore, the decrease in SRGAP2a correlated with a decrease in proteinuria and glomerular filtration rate in patients (Pan et al., 2018). They identified SRGAP2a as a podocyte-specific protein by colocalizing it with the podocyte marker synaptic regulatory protein by immunofluorescence staining (Pan et al., 2018). Inhibition of podocyte SRGAP2a significantly disrupted the podocyte cytoskeleton and enhanced podocyte migration (Pan et al., 2018). These results suggest that SRGAP2a is involved in maintaining the stability of the podocyte membrane. Mechanistically, SRGAP2a maintains podocyte stability by inactivating the GTPases RhoA and Cdc42 (Pan et al., 2018). A deficiency of Cdc42 or RhoA can lead to severe proteinuria and foot process effacement (Huang et al., 2016). Excessive activation of RhoA in podocytes also promotes the development of proteinuria by interfering with the actin cytoskeleton and focal adhesions (Zhu et al., 2011). Therefore, tightly controlled Rho-GTPase activity is essential for maintaining a stable podocyte actin cytoskeleton and normal podocyte function. The difference in the functions of SRGAP1 and SRGAP2a may be due to the binding of different Rho families of small GTPases in different cells during different pathological processes. SLIT-ROBO signaling mediated by two different SRGAPs plays different roles in podocyte stability. The role that drugs targeting the SLIT ligand and ROBO receptor will play in diabetic patients is unknown.

#### 5.4.3 FSGS

The main feature of focal segmental glomerulosclerosis (FSGS) is proteinuria associated with podocyte damage (De Vriese et al., 2018). In the previous sections, we mentioned that the SLIT2-ROBO2 signaling pathway regulates podocyte adhesion and foot process formation; therefore, inhibition of the SLIT2-ROBO2 signaling pathway may be a new target for the treatment of FSGS. PF-06730512 is a novel, recombinant, ROBO2 human immunoglobulin G1 (IgG1) crystallized fragment (Fc) fusion protein that is in clinical development for the treatment of FSGS (Lim et al., 2021). It reduces SLIT2-ROBO2 downstream signaling and improves podocyte structure and function by blocking the interaction of SLIT2 with ROBO2 (Lim et al., 2021). A clinical trial evaluated the safety, tolerability, immunogenicity, and pharmacokinetics of PF-06730512 in single or multiple escalating doses in healthy adult participants (Lim et al., 2021). This study included six single ascending dose (SAD) cohorts and four multiple ascending dose (MAD) cohorts. Adverse events occurred more frequently in the PF-06730512 group than in the placebo group (Lim et al., 2021). The most commonly reported treatment-related adverse event in the SAD cohort was headache, and the most commonly reported treatment-related adverse events in the MAD cohort were dry skin and headache (Lim et al., 2021). In all cohorts, the majority of adverse events were mild or moderate in severity (108 and 21, respectively). The incidence of immunogenicity was low, with no ADA-positive samples in the SAD cohort and two ADA-positive samples in the MAD cohort (Lim et al., 2021). In this human study, PF-06730512 was largely safe, most adverse events were mild, and the frequency of emergencies was not dose dependent (Lim et al., 2021). Immunogenicity did not occur in the majority of subjects, indicating good resistance to PF-06730512 (Lim et al., 2021). In conclusion single doses of PF-06730512 up to 1,000 mg (intravenous) and multiple doses of PF-06730512 up to 1,000 mg (intravenous) and 400 mg (subcutaneous) were safe and well tolerated in healthy participants (Lim et al., 2021). After assessing the safety and tolerability of PF-06730512, investigators evaluated the preliminary efficacy and safety of the ROBO2 fusion protein in patients with FSGS in a separate clinical trial (Beck et al., 2021). However, the results of this trial have not yet been reported. Since the ROBO2 fusion protein targets the structure and function of podocytes regulated by SLIT2-ROBO2, all diseases that can cause structural disruption of podocytes are theoretically within the therapeutic scope of PF-06730512. This study excluded other diseases that cause loss of podocyte function to assess the efficacy of PF-06730512 in FSGS patients, advancing the clinical application of PF-06730512 and providing a new target for the treatment of FSGS.

#### 5.4.4 Lupus nephritis

Systemic lupus erythematosus (SLE) is a complex, chronic inflammatory autoimmune disease that can accumulate in multiple organs (Kaul et al., 2016). One of the kidneys is often damaged. A recent clinical study identified SLIT2 as a biological marker of renal impairment in SLE (Zhang et al., 2021). They divided 103 SLE patients into four groups, namely, SLE patients with sole renal impairment (SK, *n* = 32), SLE patients with sole skin involvement (SS, *n* = 20), SLE patients who had both renal and skin impairment (KS, *n* = 18), and SLE patients with no kidney and skin involvement (NKS, *n* = 33) (Zhang et al., 2021). Patients in the SK and SS groups had higher serum SLIT2 levels than those in the NKS group, while patients in the KS group had higher serum levels than those in the SS and SK groups, suggesting that SLE patients with skin damage or kidney damage had higher levels of serum SLIT2 (Zhang et al., 2021). Further study found that serum SLIT2 levels were higher in patients with active SLE than in those with inactive SLE (Zhang et al., 2021). Furthermore, SLIT2 levels were positively correlated with anti-dsDNA antibody levels and negatively correlated with complement C4, a result that suggests a correlation between serum SLIT2 levels and SLE activity (Zhang et al., 2021). Immunofluorescence results showed high expression of SLIT2 and ROBO1 in renal tubular epithelial cells from patients with lupus nephritis (Zhang et al., 2021). This study identified an association between increased serum SLIT2 levels and activity, renal damage, and skin damage in patients with SLE, but did not specifically explore the association between SLIT2 and the mechanisms underlying the development of renal damage in SLE. Previous studies have suggested that in systemic sclerosis (SSc), an upregulation of serum SLIT2 levels promotes skin damage by disrupting endothelial cell function (Romano et al., 2018). Therefore, we speculate that in SLE, SLIT2-ROBO1 signaling is involved in SLE renal damage via an unknown mechanism.

#### 5.4.5 Renal cystic disease

Renal cystic diseases include genetic, developmental and acquired disorders (Cornec-Le Gall et al., 2014). Hereditary renal cystic disease is caused by abnormalities of the renal tubular epithelium, including defects in differentiation, polarization and cilia production, which ultimately lead to the development of renal cysts (Cornec-Le Gall et al., 2014). In a study on the mechanisms of renal cystic disease, ROBO2 deficiency was found to cause cystic kidney, where cystic cells in the renal tubular epithelium exhibit cilia defects and polarity defects (Li Q. et al., 2019). They further found that knockdown of ROBO2 caused upregulation of P53 and P21, molecules associated with cellular senescence (Li Q. et al., 2019). Previous studies have reported that phosphorylation of the E3 ubiquitin ligase MDM2 mediates the degradation of P53 and that dephosphorylation of MDM2 stabilizes P53 (Marine and Lozano, 2010). Therefore, the researchers considered whether the deletion of ROBO2 could affect the phosphorylation of MDM2. In a deepening mechanistic study, it was found that Baiap2, a multistructural domain protein, can bind to ROBO2, forming a protein complex (Li Q. et al., 2019). Baiap2 and ROBO2 are required to maintain the phosphorylation of MDM2, as deficiency of ROBO2 or Baiap2 resulted in dephosphorylation of MDM2 and upregulation of P53 expression (Li Q. et al., 2019). Overall, ROBO2 mediates the degradation of P53 by interacting with Baiap2 to maintain the phosphorylation of MDM2 and the development of renal tubular epithelial cells. Interestingly, however, the role of ROBO2 in renal cystic disease is not regulated by SLIT2, as SLIT2 does not affect MDM2 phosphorylation when ROBO2 is deficient (Li Q. et al., 2019). This suggests that ROBO receptors may function independently of the ligand SLIT, which also suggests new insights and ideas for subsequent studies of the SLIT-ROBO signaling pathway.

#### 5.4.6 Renal cell carcinoma

The development of renal cell carcinoma (RCC) is thought to be associated with the epigenetic inactivation of multiple tumor suppressor genes (TSGs) (Ibanez de Caceres et al., 2006). SLIT2 has been reported to act as a tumor suppressor in a variety of tumors (Singh et al., 2007). Promoter methylation is the main mechanism by which *Slit2* expression is downregulated in these cancers, and hypermethylation of the *Slit2* promoter region has been detected in numerous cancers (Dallol et al., 2003; Singh et al., 2007; Alvarez et al., 2013). The main focus here is on the altered expression of SLIT2 in renal cell carcinoma and the possible mechanisms. A study found that the protein levels of SLIT2 were significantly reduced in renal cell carcinoma, with a possible mechanism being hypermethylation of the promoter region of the *Slit2* gene, as the protein levels of SLIT2 were restored following the use of methylation inhibitors (Ma et al., 2014). SLIT2 may therefore play a role in inhibiting tumor progression in renal cell carcinoma. Continued in-depth study of the mechanism of SLIT2 development in renal cell carcinoma will help to understand renal cell carcinogenesis and further search for new therapeutic strategies for renal cell carcinoma.

## 6 Conclusion

SLIT ligands and their major receptor ROBO were initially discovered in the developing neural structure and are involved in axonal repulsion and neuronal migration. With further research on this signaling pathway, its actions in nonneural areas, such as cell migration, angiogenesis, tumor progression and modulation of bone metabolism, have gradually been discovered. In addition to their key roles in the developing kidney, SLIT and ROBO family members have been found to be highly expressed in the glomeruli and tubules of the mature kidney in recent years, especially in kidney tubular epithelial cells, glomerular endothelial cells, and podocytes. The special location must have a specific function, and the expression of the SLIT-ROBO molecule in mature kidneys suggests its specific role in various renal pathophysiological processes. The SLIT-ROBO signaling pathway can regulate actin cytoskeleton rearrangement by inhibiting the activity of small Rho GTPase family proteins. Reshaping of the actin cytoskeleton is involved in the migration of renal inflammatory cells, the development of fibrosis, the formation of the normal structure of podocytes, and their adhesion to the basement membrane.

In this review, we illustrate the role of SLIT-ROBO signaling in a variety of renal diseases. For example, SLIT2 improved renal function in IRI mouse models by inhibiting the adhesion of neutrophils to endothelial cells; fusion proteins targeting ROBO2 are in clinical trials in FSGS patients; and serum SLIT2 levels are a new biological indicator of kidney damage in SLE. Deletion of the *ROBO2* gene promoted the occurrence of renal cystic diseases. As a tumor suppressor gene, *SLIT2* inhibits the occurrence of renal cell carcinoma.

Although research on the SLIT-ROBO signaling pathway in the kidney has a certain foundation, other functions of the pathway in the kidney remain to be further explored. Current studies lack clinical data on this component, making the SLIT-ROBO signaling pathway particularly important in future studies in renal clinical disease. In light of the existing studies, the SLIT-ROBO pathway has great application prospects and therapeutic potential in various physiological and pathological processes of the kidney. Hence, there is a need to probe further into the contribution of SLIT-ROBO to the pathogenesis of various renal diseases.
